# Critical androgen-sensitive periods of rat penis and clitoris development

**DOI:** 10.1111/j.1365-2605.2009.00978.x

**Published:** 2010-02

**Authors:** Michelle Welsh, David J MacLeod, Marion Walker, Lee B Smith, Richard M Sharpe

**Affiliations:** MRC Human Reproductive Sciences Unit, Centre for Reproductive Biology, The Queen’s Medical Research InstituteEdinburgh, UK

**Keywords:** androgens, clitoris, flutamide, hypospadias, masculinisation programming window, micropenis, penis length

## Abstract

Androgen control of penis development/growth is unclear. In rats, androgen action in a foetal ‘masculinisation programming window’ (MPW; e15.5–e18.5)’ predetermines penile length and hypospadias occurrence. This has implications for humans (e.g. micropenis). Our studies aimed to establish in rats when androgen action/administration affects development/growth of the penis and if deficits in MPW androgen action were rescuable postnatally. Thus, pregnant rats were treated with flutamide during the MPW ± postnatal testosterone propionate (TP) treatment. To assess penile growth responsiveness, rats were treated with TP in various time windows (late foetal, neonatal through early puberty, puberty onset, or combinations thereof). Phallus length, weight, and morphology, hypospadias and anogenital distance (AGD) were measured in mid-puberty (d25) or adulthood (d90) in males and females, plus serum testosterone in adult males. MPW flutamide exposure reduced adult penile length and induced hypospadias dose-dependently; this was not rescued by postnatal TP treatment. In normal rats, foetal (e14.5–e21.5) TP exposure did not affect male penis size but increased female clitoral size. In males, TP exposure from postnatal d1–24 or at puberty (d15–24), increased penile length at d25, but not ultimately in adulthood. Foetal + postnatal TP (e14–postnatal d24) increased penile size at d25 but reduced it at d90 (due to reduced endogenous testosterone). In females, this treatment caused the biggest increase in adult clitoral size but, unlike in males, phallus size was unaffected by TP during puberty (d15–24). Postnatal TP treatment advanced penile histology at d25 to more resemble adult histology. AGD strongly correlated with final penis length. It is concluded that adult penile size depends critically on androgen action during the MPW but subsequent growth depends on later androgen exposure. Foetal and/or postnatal TP exposure does not increase adult penile size above its ‘predetermined’ length though its growth towards this maximum is advanced by peripubertal TP treatment.

## Introduction

Formation of a penis is induced by androgen action in the first trimester of pregnancy in humans (Husmann, 2002), and most of its subsequent growth is also androgen-dependent (Husmann, 2002; Boas *et al.*, 2006; Camurdan *et al.*, 2007). Therefore normal length/size of the penis depends critically on androgens, but how and when androgens mediate this is unclear. This is particularly true if a penis is normally formed but abnormally small (micropenis) or is malformed as in hypospadias (Bin-Abbas *et al.*, 1999; Husmann, 2002). Penis size amongst normal men varies considerably (Ponchietti *et al.*, 2001) and men’s anxiety about ‘normality’ of their penis size is widespread (Vardi, 2006). Clarification of when androgens act to promote normal penis formation and growth should therefore advance understanding of causes of abnormal penile development/growth and its management.

Manifestation of micropenis at birth in boys is interpreted as there having been deficient androgen action during late gestation (Bin-Abbas *et al.*, 1999; Husmann, 2002; Grumbach, 2005). Boys with micropenis and some with hypospadias are frequently treated in the first few months with testosterone to induce penile growth. Whilst this is generally successful (Bin-Abbas *et al.*, 1999), a proportion show a poor growth response (Husmann, 2004; Lee & Houk, 2004; Zenaty *et al.*, 2006) and most boys with micropenis ultimately have a smaller than average penis in adulthood (Husmann, 2002; Lee & Houk, 2004). Studies in rats have also questioned whether neonatal/infant testosterone treatment of micropenis might impair androgen-dependent growth during puberty (Husmann & Cain, 1994; Levy *et al.*, 1996). Although current consensus is that testosterone treatment does not impair later penis growth (Sutherland *et al.*, 1996; Baskin *et al.*, 1997; Bin-Abbas *et al.*, 1999) doubts persist about optimal timing of treatment (Lee & Houk, 2004) mainly as a result of poor understanding of penis development.

Recent studies in rats have identified a ‘masculinisation programming window’ (MPW) at the onset of foetal testosterone production (e15.5–e18.5), when sufficient androgen action must occur to ensure normal formation (and subsequent development) of the penis and reproductive tract (Clark *et al.*, 1993; Welsh *et al.*, 2008). Subnormal androgen action during the MPW may induce hypospadias and results in a smaller than normal penis at puberty (Welsh *et al.*, 2008). In comparison, blocking androgen action in late gestation may affect penile growth (Husmann, 2002) but does not induce hypospadias (Welsh *et al.*, 2008). Therefore penis formation and growth are both androgen-dependent, but are affected by androgens in different time windows, the ‘formation window’ (MPW) being narrow, whereas the ‘growth window’ is wide. Evidence points towards a similar MPW in humans (probably within 8–14 weeks’ gestation; Welsh *et al.*, 2008) and in non-human primates (Prahalada *et al.*, 1997; Herman *et al.*, 2000). Androgen-dependent penile growth occurs at three time points in humans: in late gestation (Husmann, 2002; Grumbach, 2005), the first 4 years after birth (Husmann, 2002; Grumbach, 2005; Boas *et al.*, 2006; Camurdan *et al.*, 2007) and at puberty (Husmann, 2002). However, although androgens regulate penile growth postnatally, androgen action during the MPW may determine the capacity for this growth (Welsh *et al.*, 2008).

If androgen action within the MPW determines formation of a normal penis and its size, then this implies that subnormal androgen action during the MPW cannot be rescued by later androgen action (Welsh *et al.*, 2008). This could explain the poor penile growth response and below average adult penile size in some boys with micropenis (Husmann, 2004; Lee & Houk, 2004; Zenaty *et al.*, 2006); it might also explain some of the variation in penile size amongst normal men. Therefore, the aim of this study in rats was to establish when androgen-dependent growth of the normal and abnormal penis is induced and how this relates to final penile length.

## Materials and methods

### Animals and treatments

**Figure 1:**
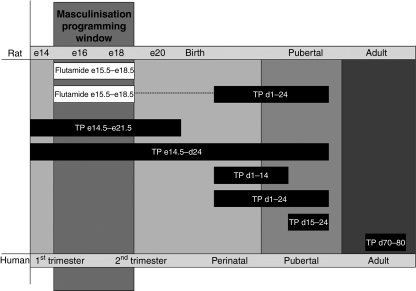
Schematic diagram to show timings of rat treatments in the current studies and their relation to comparable timings in humans. The masculinisation programming window in rats occurs between e15.5–e18.5 and is predicted to occur in humans between weeks 8–14.

Wistar rats were maintained under standard conditions according to UK Home Office guidelines. Animals had free access to water and a soy-free breeding diet (SDS; Dundee, Scotland). Time-matings were established and presence of a vaginal plug was defined as embryonic day 0.5 (e 0.5); for this study at least 3 L were used per treatment group. Treatment regimes are summarised in Fig. 1. In the flutamide (Sigma-Aldrich, Poole, UK) studies, dams were dosed daily from e15.5 to e18.5 by oral gavage with 0, 2, 5, 10 or 100 mg/kg in 1 mL/kg corn oil/2.5% DMSO (Sigma-Aldrich); this treatment time window encompasses the MPW (Welsh *et al.*, 2008).

As flutamide exposure in the MPW reduces adult penile size, we investigated if exogenous postnatal androgens could overcome this deficit. Thus, 9 flutamide-exposed (10 mg/kg) male offspring were treated postnatally from d1 to d24 with 20 mg/kg testosterone propionate (TP; Sigma-Aldrich) and examined on d90 (adulthood); this treatment window for TP was selected as it was the most effective for increasing penile growth in the controls (see Results).

For prenatal TP treatment, pregnant dams were injected subcutaneously with 20 mg/kg TP in 0.4 mL corn oil daily from e14.5–e21.5. This treatment can induce dystocia (Welsh *et al.*, 2008) so to avoid foetal mortality and maternal suffering, foetuses from TP-treated dams were caesarian-derived and cross-fostered to untreated mothers that had delivered within the previous 6 h. For postnatal TP treatment, pups were injected subcutaneously with 20 mg/kg TP in 0.4 mL corn oil every third day from d1–14, d1–24 or d15–24. Some animals exposed prenatally to TP were also treated postnatally with TP. Details of animal numbers are shown in Table 1. In one experiment, adult male rats (d70) were treated for 10 days with 20 mg/kg TP according to the above regime and examined on d80.

**Table 1:** Numbers of animals treated during the various time windows with vehicle (controls) or with testosterone proprionate (TP) and their sampling ages

	Postnatal day 25 (mid-puberty)		Postnatal day 90 (adulthood)		Adult serum testosterone (ng/mL)
Postnatal TP-treatment group	Males	Females	Males	Females	Males (day 90)
Controls (vehicle)	18	20	12	11	2.33 ± 0.45
TP e14.5–e21.5	8	7	9	3	4.10 ± 0.45
TP e14.5–d24	8	8	6	6	0.15 ± 0.02***
TP d1–d24	9	6	7	5	3.50 ± 1.75
TP d15–d24	5	5	4	5	3.70 ± 0.98

Serum levels of testosterone (means ± SEM) in males at adulthood (d90) are also shown.

*** *p*< 0.001, in comparison with the respective control group.

### Tissue recovery and penile measurements

Animals were killed on d25 or d90 by inhalation of carbon dioxide and cervical dislocation. Blood was collected by cardiac puncture and plasma testosterone and luteinising hormone (LH) concentrations were measured as previously published (Atanassova *et al.*, 1999; Welsh *et al.*, 2008). Anogenital distance (AGD) was measured using digital callipers (Faithfull Tools, Kent, UK) as a measure of androgen exposure during the MPW (Welsh *et al.*, 2008). Normal penile morphology was assessed using previously established criteria (Welsh *et al.*, 2008). The phallus from both sexes was dissected out, its length measured with digital callipers and weighed, fixed for 6 h in Bouins, transferred into 70% ethanol and processed into paraffin wax (Welsh *et al.*, 2008).

### Penile histology

Penis histology was examined using Goldner’s stain on penile cross-sections from animals in each of the treatment groups (Welsh *et al.*, 2008). Penises from d90 animals were decalcified by incubating in neutral EDTA for 10 days at 37 °C prior to sectioning/staining.

### Statistical analysis

Data were analysed using graphpad prism version 5 (Graph Pad Software Inc., San Diego, CA, USA) and one-way analysis of variance (anova) followed by the Bonferroni post-test. Incidence of hypospadias was analysed using Fisher's exact test. Correlation between AGD and penile length was analysed using linear regression.

## Results

### Prenatal androgen action determines adult penile length in males

**Figure 2:**
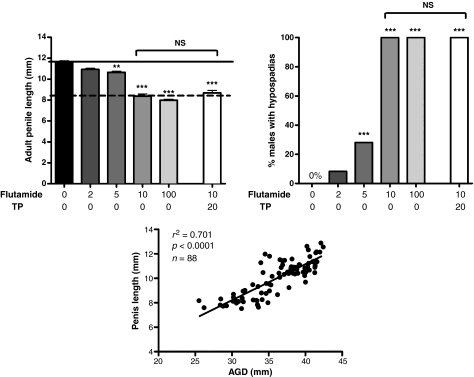
Penile length (means ± SEM; top left), incidence of hypospadias (top right) and the relationship between penile length and anogenital distance (AGD; bottom) in adult male rats that had been exposed in utero (e15.5–e18.5) to vehicle (controls) or various doses of flutamide (left) and the effects of postnatal administration of testosterone propionate (TP) from d1 to d24 (right) in a group of animals prenatally exposed to 10 mg/kg flutamide. Values in the upper left panel are means ± SEM for 9–20 animals per group (overall n = 88). Solid horizontal line shows the mean penile length for control males and the dashed horizontal line shows the mean for males exposed in utero to 10 mg/kg flutamide. **p< 0.01, ***p< 0.001, in comparison with the control group. Postnatal TP treatment had no significant effect (NS) on penile length in comparison with animals prenatally exposed to flutamide alone.

Treatment of pregnant females with flutamide during the MPW (e15.5–e18.5) caused a dose-dependent decrease in adult penile length in male offspring, independent of the occurrence of hypospadias (Fig. 2). Postnatal TP treatment after foetal flutamide (10 mg/kg) exposure did not alter adult penile length (Fig. 2) in comparison with the corresponding flutamide-exposed, non-TP-treated animals (Fig. 2). Adult penile length was highly correlated with AGD in the control and flutamide-treated males (Fig. 2).

### Effect of TP administration to the controls on penile length in males

**Figure 3:**
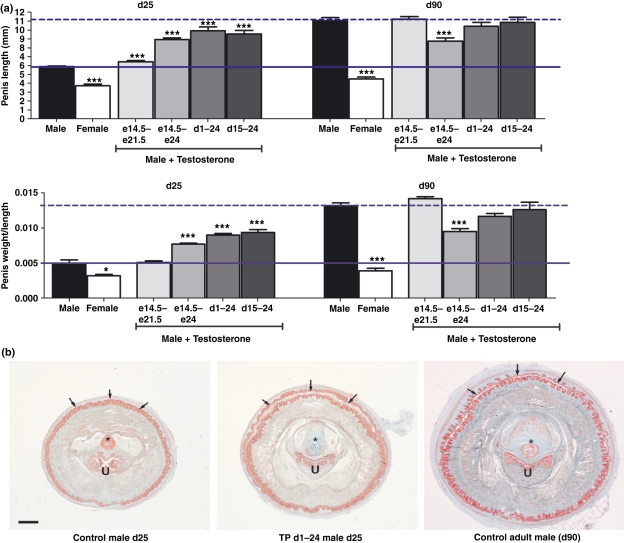
(a) Penile length (top panels) and weight/length (lower panels) at mid-puberty (d25) or in adulthood (d90) in male rats exposed in utero (e14.5–e21.5) or postnatally from d1 to d24 or from d15 to d24, or a combination of prenatal and postnatal exposure (e14.5–d24), to testosterone propionate (TP). Data are shown for vehicle-treated males (controls) and females for comparison. Solid horizontal line shows the mean penile length for control males at d25 and the dashed horizontal line shows the mean for d90 male controls. *p< 0.05, **p< 0.01, ***p< 0.001, in comparison with the respective control group. Values are means ± SEM for the number of animals shown in Table 1. (b) Representative penile cross-sectional morphology in the control rats at d25 (left) and d90 (right) in comparison to a d25 rat treated postnatally from d1 to d24 with TP (middle). In the latter, note the increased penile diameter, preputial separation (small arrows) and ossification of the os penile bone (*blue-green staining) in comparison with the d25 control, and its advancement towards the adult phenotype. Sections were stained with Goldners as described elsewhere (Welsh *et al.*, 2008). Scale bar shows 0.5 mm. U, urethra.

Testosterone propionate exposure in any time window significantly increased penile length in males at d25 compared with vehicle (Fig. 3a). This enhancement was the smallest for foetally TP-exposed males and greatest for those exposed postnatally from d1 to d24 (Fig. 3a). None of these enhancements persisted into adulthood, as TP treatment at any time failed to augment adult penile length (Fig. 3a). Indeed, combined prenatal + postnatal TP treatment (e14.5–d24) resulted in decreased ultimate adult penile length (Fig. 3a), presumably because of the subnormal endogenous testosterone levels in this treatment group (<10% of the control levels; Table 1). In turn, this decrease in endogenous testosterone concentrations is probably explained by abnormally low LH concentrations in this group (controls 4.2 ± 0.06 ng/ml, *n* = 8; TP e14.5–d24 0.6 ± 0.09, *n* = 6; *p*< 0.001). Testosterone levels were normal in all other TP-treated groups (Table 1). Similar results were found for penile weight/length (as an indirect measure of girth) as for length (Fig. 3a) in males at d25 and d90.

Comparison of penile length at d25 and d90 showed no significant change in penile length between d25 and d90 in groups that included postnatal TP treatment, whereas vehicle-treated controls showed *a* > 80% increase (*p*< 0.001) in penile length over this period (Fig. 3a). This implies that postnatal TP treatment simply advances penile growth, rather than enhancing it. This was confirmed by examination of penile morphology of the group with the greatest advancement of penile growth at d25 (i.e. TP d1–24). This showed normal gross morphology but with increased penile diameter, ossification of the os bone and separation of the prepuce, all of which represent a precocious advance towards the normal adult phenotype (Fig. 3b). Consistent with this, TP administration to adult rats for 10 days did not increase penile length (controls 11.0 ± 0.58 mm, *n* = 4; TP 11.1 ± 0.27, *n* = 6; means ± SEM).

### Effect of TP administration on clitoral length/growth in females

**Figure 4:**
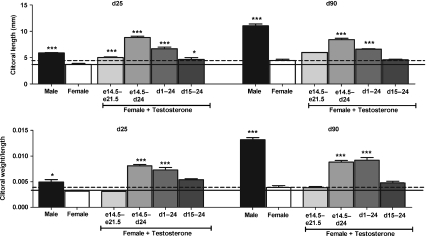
Clitoral length (top panels) and weight/length (lower panels) at mid-puberty (d25) or in adulthood (d90) in female rats exposed in utero (e14.5–e21.5) or postnatally from d1 to d24 or from d15 to d24, or a combination of prenatal and postnatal exposure (e14.5–d24), to testosterone propionate (TP). Data are shown for vehicle (controls)-treated males and females for comparison. Solid horizontal line shows the mean clitoral length for control females at d25 and the dashed horizontal line shows the mean for d90 female controls. *p< 0.05, ***p< 0.001, in comparison with the respective control group. Values are means ± SEM for the number of animals shown in Table 1.

Foetal exposure (e14.5–e21.5) of females to TP caused a small increase in clitoral length at d25, but maximum clitoral length occurred with combined foetal and postnatal treatment (e14.5–d24; Fig. 4). TP treatment from d1 to 24 increased clitoral length at d25 whereas treatment from d15 to 24 caused a notably smaller increase (Fig. 4). In all TP treatment groups, clitoral length at d90 remained comparable with the length at d25 whereas the controls showed a modest size increase from d25 to d90 (Fig. 4).

## Discussion

The primary aim of these studies was to determine when penile length and growth are determined by androgens in the rat. This was prompted by the data from clinical studies involving testosterone treatment of boys with micropenis and/or hypospadias and the uncertainty over whether the age of testosterone treatment (of micropenis) affects treatment success in terms of penile growth. Earlier studies by ourselves and others have demonstrated that androgen-driven growth/elongation of the rat penis continues after the MPW (Husmann & Cain, 1994; Levy *et al.*, 1996; Welsh *et al.*, 2008), consistent with current understanding in humans and non-human primates (Liu *et al.*, 1991; Brown *et al.*, 1999; Herman *et al.*, 2000; Husmann, 2002; Grumbach, 2005; Boas *et al.*, 2006). This raised the possibility that testosterone administration to rats for a sufficient period postnatally might restore normal penile length in foetally flutamide-exposed rats or increase ultimate penis size in the control animals. Such studies would assist in management of boys with penile abnormalities.

Our results show that adult penile size in rats requires adequate androgen action within the MPW and that deficiencies in androgen action at this time ‘dose-dependently’ reduces penile length in adulthood, extending our previous findings (Welsh *et al.*, 2008). This deficit cannot be rescued by postnatal TP treatment, even though androgens increase penile growth in the neonatal and peripubertal periods in rats (this study; Husmann & Cain, 1994; Levy *et al.*, 1996; Husmann, 2002). Our findings show that postnatal testosterone treatment of rats at any stage prior to achievement of adult penile size (Fig. 1) will advance, but not ultimately enhance, penile size, consistent with earlier findings (Levy *et al.*, 1996). These conclusions derive from administration of supranormal testosterone doses to *normal* animals, so extrapolation to boys with micropenis is not straightforward. However, similar conclusions were reached in rats in which micropenis had been induced prior to testosterone treatment (Levy *et al.*, 1996) as well as after foetal flutamide treatment (this study). Androgen treatment of boys/infants with micropenis suggests that the human penis is responsive to growth stimulation by exogenous androgens at comparable stages of development to rats (Bin-Abbas *et al.*, 1999; Grumbach, 2005).

Based on our findings, it is likely that final penile size in men depends on a sufficient level of androgen action within the MPW and that deficient androgen action at this time will have permanent and irrecoverable consequences for final penile size. In this regard, boys with isolated micropenis show a bigger increase in penile length in response to testosterone treatment than do boys with micropenis + hypospadias (Velasquez-Urzola *et al.*, 1998). This fits with our findings which show that hypospadias in rats arises as a result of the deficient androgen action only within the MPW (Welsh *et al.*, 2008) and such males have reduced adult penile length and growth capacity (this study). This being the case, an important question is can penile growth capacity be predicted at birth? Our studies show that AGD in adulthood (this study) and in puberty (Welsh *et al.*, 2008) correlate highly with penile length, consistent with both being determined by androgen action within the MPW (Welsh *et al.*, 2008). Distinctive differences in male–female AGD are evident at birth in humans (Swan *et al.,* 2005; Swan, 2008) as in rats (McIntyre *et al.*, 2001), and reduced AGD is associated with hypospadias in both (Hsieh *et al.*, 2008; Welsh *et al.*, 2008). Therefore, measuring AGD at birth may predict the growth capability and adult size of the penis in humans because AGD at birth and adulthood are highly correlated in rats (McIntyre *et al.*, 2001) and adult AGD is related to final penile length (this study). Two studies have reported a positive relationship between AGD (corrected for bodyweight) and penis size/length in infant boys (Swan *et al.,* 2005; Swan, 2008) whereas another study found no correlation (Romano-Riquer *et al.*, 2007), although this study did not correct AGD for bodyweight, an important confounder in babies (Swan, 2008).

The previous studies (Jacobs *et al.*, 1977; Levy *et al.*, 1996) showed that testosterone administered to rats peripubertally or in adulthood in similar/higher doses to those used in these studies, did not enhance adult penile size. Our findings, involving testosterone exposure in a wider range of time-windows, confirm this. Moreover, foetal androgen exposure during or during and after the MPW (Welsh *et al.*, 2008) does not enhance ultimate penile size, showing that unknown non-androgenic factors must predetermine the maximum size to which the penis can grow (‘penile potential’). We suggest that this is the first of three stages in penis development. Androgen action is essential to achieve this potential, most critically within the MPW (stage 2), but also in late foetal life and postnatally when growth of the penis to its predetermined level depends on sufficient androgen stimulation (stage 3). In normal animals, testosterone levels in foetal and postnatal life are sufficient to complete stages 2 and 3. Deficiencies in androgen action in stage 3 are potentially recoverable by later androgen treatment, whereas deficiencies in stage 2 are not. Our data (based on androgen action) also suggests that final penile size cannot be increased therapeutically in normal males.

Earlier rat studies suggested that testosterone treatment in some postnatal periods might be detrimental to final penis size (Husmann & Cain, 1994; Levy *et al.*, 1996; Husmann, 2002). Our study shows that TP treatment spanning birth (foetal + postnatal) led to significantly reduced penile size in adulthood, whereas TP treatment either foetally or postnatally did not have this effect. These findings can be explained by the near baseline levels of testosterone found in adulthood in the foetal + postnatal TP-treatment group, because of grossly suppressed LH levels, indicating failure to establish a normally functioning hypothalamic–pituitary–testicular axis. The latter is programmed around birth in rats (Handa *et al.*, 1985) and has presumably been disrupted by the exogenous androgen exposure. This further illustrates the importance of endogenous androgens for the adult penis to grow to its predetermined length.

The effect of testosterone exposure on clitoral development in females was investigated as a comparison to males. Our results show that clitoral growth in females remains androgen-responsive after birth, but for a more restricted period than the penis in males, as unlike males there was little responsiveness to TP at d15–24. In human females, the clitoris is androgen growth-responsive foetally and postnatally (Sane & Pescovitz, 1992) and even in adulthood (Slayden, 1998; Gooren & Giltay, 2008), perhaps suggesting some difference from rats. An interesting observation from this study is that TP treatment from d1 to 24 induced a substantial increase in clitoral size that was maintained through to adulthood despite the cessation of TP treatment after d24. Combined foetal and postnatal (d1–24) androgen exposure caused an even larger, permanent increase in clitoral size.

In conclusion, we show that adult penile size in rats is critically dependent on exposure to sufficient androgen action during the foetal MPW. Deficiencies in androgen action in the MPW are irrecoverable by postnatal androgen treatment, even though the penis remains androgen growth-responsive postnatally. Assuming the same applies to humans, it may explain the limited growth of the penis that occurs after androgen treatment of some boys with micropenis. Based on the relationship between AGD and final penile length in rats, penile growth responsiveness may be predictable by measuring AGD of boys at birth. Finally, additional androgen exposure at any age in rats cannot enhance ultimate penile length, although it can advance growth postnatally towards its predetermined maximum. The latter is determined in utero by unknown androgen-independent factors, but sufficient androgen action (in the right time windows) is necessary to achieve this potential.
